# The interplay of family capital and learning engagement: Mediating mechanisms in undergraduates’ intentions to take the postgraduate entrance examination

**DOI:** 10.1371/journal.pone.0339529

**Published:** 2026-01-09

**Authors:** Chen Xiaoman, Zhou Hongfang

**Affiliations:** 1 School of Educational Sciences, Sichuan Normal University, Chengdu, Sichuan, China; 2 School of Marxism, Xihua University, Chengdu, Sichuan, China,; 3 School of Marxism, Sichuan Normal University, Chengdu, China; Zhejiang Normal University, CHINA

## Abstract

**Background:**

The phenomenon of “postgraduate examination fever” has raised concerns about the factors influencing undergraduates’ decisions to pursue graduate education. By conducting a cross-sectional survey of 1,178 undergraduates from Double First Class and ordinary universities in China, this study examines how family capital and learning engagement jointly affect Chinese undergraduates’ intentions to take the national postgraduate entrance examination under theories of capital and student engagement.

**Methods:**

This study used a bootstrapped mediation analysis, two stage sampling, convenience sampling, and a binary logistic regression model to investigate the impact of these variables on the intention to pursue graduate education in order to ascertain whether learning engagement mediates the impact of family capital among undergraduates from both Double First Class and ordinary universities in China.

**Results:**

This study reveals that both family capital and learning engagement were significant predictors of students’ intention to pursue graduate studies (p < 0.01). Learning engagement had a notably stronger effect than family capital in the logistic model, odds ratio for learning engagement = 1.62, and 1.20 for family capital, although both were significant. Moreover, family capital had a significant indirect effect on intention through learning engagement, indicating a partial mediating mechanism.

**Implication:**

The significance of this study lies in its demonstration that family capital influences postgraduate aspirations indirectly through the enhancement of learning engagement, thereby enriching the explanatory power of educational reproduction theories. Furthermore, it offers practical and policy implications by highlighting that narrowing resource disparities while simultaneously fostering student engagement can effectively promote both educational equity and academic advancement.

## 1 Introduction

Across many developing systems of higher education, massification has expanded university access while compressing advantages once conferred by an undergraduate credential, China is emblematic. Official forecasts reported that the number of college graduates would reach an unprecedented 13.56 million in 2024, intensifying competition for graduate, track jobs and fueling a widespread perception of credential inflation [1]. One prominent response has been the surge in applications for the national postgraduate entrance examination, widely discussed as a persistent “postgraduate examination fever” in public and policy discourse [2]. Understanding why undergraduates intend to pursue postgraduate study in this context is therefore both an empirical necessity and a policy priority.

A growing body of research centers family capital as a key determinant of both learning during college and postcollege willingness. Studies document how familial expectations, cultural knowledge, and material support structure students’ understandings of graduate school, lower subjective costs, and sustain aspirations, with particular salience for first generation and minoritized students [3,4]. Research indicates that familial resources are associated with increased learning engagement in college, and that high relevant engagement experiences connect with a greater intention to pursue postgraduate studies [5]. Educational interventions can impact final year students, so indirectly affecting their participation and desires for continued education [6]. Collectively, these elements provide a theoretically consistent framework wherein familial capital facilitates learning engagement.

Despite important advances, two gaps remain, especially in the Chinese context of massification and credential competition. First, much research examines either family background or engagement but rarely specifies and tests mediating mechanisms that connect family capital, learning engagement, postgraduate willingness within a single model anchored in established theory [7,5]. Second, evidence on the family capital, learning engagement, postgraduate willingness’ nexus remains dominated by western settings. China’s institutional logics may condition these relationships in distinctive ways that are under documented. Responding to these gaps, this study focuses on undergraduates’ intentions to take China’s postgraduate entrance examination.

## 2 Literature review

In addition to macroeconomic factors like the labor market, the rise in graduate student enrollment is intricately linked to microlevel influences, including family and individual factors. Coleman’s seminal report has underscored the significance of family capital in shaping college students’ educational trajectories [8]. At the same time, scholars, focusing on family capital, further education decision making process and learning engagement of college students, have conducted extensive research and reached several conclusions.

(1) Family capital affects the decision making process of undergraduate students’ education [9]. Qualitative research shows that family related motives including intergenerational uplift, honoring familial expectations, and drawing on family knowledge, are salient for students who aspire to graduate school, particularly among women of color and first generation students [3,4]. Studies of Hispanic or Latinx STEM majors similarly highlight how familial and community cultural wealth facilitate graduate application [10]. Emerging evidence in STEM also points to the formative role of family, alongside peers and mentors, in shaping motivational beliefs and career pathways linked to postgraduate decisions [11].

Consistent with rational choice accounts, the education decision is structured by direct and opportunity costs like fees, living expenses, foregone earnings, subjective success probabilities, and anticipated benefits. Variation in family capital alters these trade-offs and the strategies students consider viable [7]. In the UK context, evidence suggests that while debt levels can matter, underlying financial resources and attainment are more predictive of progression to postgraduate study than debt itself [12,13]. Large scale and China based studies further indicate that family cultural and economic capital are positively associated with access to higher education and with academic outcomes that underpin postgraduate eligibility, with notable heterogeneity by social strata and institutional field [14–16].

At the same time, a growing literature argues for intersectional and context sensitive perspectives, showing that family influences operate through multiple forms of capital like aspirational, familial, navigational, are refracted by institutional settings and student identities [3,16]. Building on this evidence base, this study proposes H1.

**H1. Family capital positively influences undergraduates’ willingness to pursue postgraduate study.**

(2) The impact of family capital on undergraduate students’ learning engagement.

A growing empirical base links family capital to engagement in higher education. Structural equation studies with Chinese undergraduates, for example, show that family capital predicts higher learning engagement and that engagement partially mediates effects on subsequent gains [17]. Complementary evidence connects family socioeconomic status to engagement via psychosocial pathways like resilience, future orientation, autonomy support, suggesting that family resources and the motivational climate of the home jointly shape how students invest effort in learning [13]. Qualitative research involving students from low income backgrounds similarly examine how familial expectations and support, or their lack thereof, influence daily study practices and persistence [18]. Taken together, these studies are consistent with the “educational interface” account in which structural and psychosocial influences many of them family based, work through the mechanisms that generate engagement [5].

At the same time, effects are heterogeneous across contexts and student groups. Work distinguishing rural and urban settings finds that family cultural and social capital differentially predict academic effort and aspiration by hukou type, underscoring that the same “capital” travels unevenly across institutional and socio geographic ecologies [14]. Reviews also flag conceptual and methodological fragmentation, variable operates of “family capital” cross sectional designs, convenience samples, and call for more longitudinal and qualitative designs capable of tracing how multiple capital forms interact with institutional settings and student agency to shape engagement over time [19,20]. These gaps matter because estimates of “family capital effects” on engagement are likely to be design sensitive and context contingent. Building on this evidence base, this study proposes H2.

**H2. Family capital positively influences undergraduates’ learning engagement.**

(3) Research on undergraduate students’ learning engagement and their willingness for postgraduate study. Research on students’ educational investment decisions has shifted from purely structural accounts to a more integrative view that combines psychological motivations, financial capability, socio-economic background, and institutional context. At the individual level, studies consistently show that intrinsic and extrinsic reasons, such as the desire to learn, career advancement, social purpose, role modeling, and mobility, shape willingness to invest in further study [21,22]. Attitudes toward postgraduate study and perceived social expectations are especially salient: work grounded in the Theory of Planned Behavior finds that attitudes and norms predict the intention to pursue a master’s degree, net of background factors [23,24]. Within a rational choice tradition, students reweigh expected benefits, costs and perceived probabilities of success, with these parameters structured by classed resources and information [7]. Emerging evidence also indicates that financial literacy and mandated personal finance education can shift financing choices toward lower cost aid, altering the perceived affordability of postgraduate routes [25]. Taken together, these strands situate learning engagement, the intensity and quality of students’ cognitive, behavioral, and affective involvement in study, as the proximal mechanism translating motivations, capabilities, and contexts into concrete continuation intentions.

Conceptual frameworks of student engagement argue that engagement emerges at the “educational interface” where student characteristics and institutional affordances interact to shape self-efficacy, belonging, and emotions that, in turn, influence achievement and forward looking choices [5]. Empirically, forms of high impact engagement such as undergraduate research are associated with stronger intentions to apply to and enroll in graduate programs, partly through strengthened science/academic identity and mentoring relationships [26]. Expectancy value theory provides a complementary account: students engage more deeply when they expect success and perceive high task value; these same appraisals also predict persistence and subsequent enrollment choices [27]. Thus, learning engagement is not only a correlate of academic outcomes; it is a plausible antecedent of postgraduate intentions because it amplifies perceived competence, value, and goal alignment. Building on this evidence base, this study proposes H3.

**H3. Undergraduates’ learning engagement positively predicts their intention to pursue postgraduate study.**

Family capital shapes both the resources available for study and the informational and normative environments students inhabit. Comparative and country specific evidence shows that family background and parental education indirectly influence postgraduate choices via academic performance and institutional access, consistent with complex mediation [28,29]. Meta analytic and longitudinal work further links socio-economic inputs to engagement processes, while engagement itself predicts downstream academic behaviors and choices [5]. Building on these strands, it is theoretically coherent to model learning engagement as a mediator between family capital and willingness to pursue postgraduate study: family capital enables engagement through material support, cultural know how, and networks; engagement then heightens perceived success probabilities and value, translating background advantage into continuation intentions. Building on this evidence base, this study proposes H4.

**H4. Learning engagement mediates the relationship between family capital and intention to pursue postgraduate study.**

## 3 Construction of the research framework

**Fig 1:**
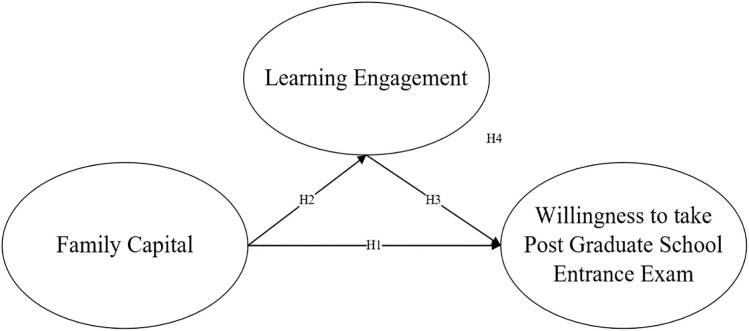
Theoretical model of family capital, study engagement and undergraduate students’ willingness to enter graduate school.

In order to explore the relationship between family capital, study engagement and willingness to go to graduate school, this paper selects Boudon’s “primary effect” and “secondary effect” analysis theory and Goldthorpe’s related risk aversion theory for analysis. The “primary effect” refers to the influence of factors such as undergraduate students’ learning engagement, academic performance and academic performance on future education, while the “secondary effect” refers to the economic status of family capital, parents’ education level, social resources and other factors that will also affect whether college students are willing to continue to receive high level education in the future [2]. Boudon’s “primary effect” and “secondary effect” analysis theory establishes the relationship between family capital and willingness to go to graduate school, and between learning engagement and willingness to enter graduate school. Goldthorpe’s relative risk aversion theory suggests that in social mobility, each class will take corresponding measures to ensure that college students have an advantage in the process of social mobility and avoid falling behind other groups in the same class. Specifically, parents in the family will spend a lot of material and financial resources to train their undergraduate students’ education to ensure that their undergraduate students’ academic performance does not lag behind other students, and to ensure that their college students are in a comparative advantage in the process of going on to higher education. Goldthorpe’s related risk aversion theory establishes the relationship between household capital and learning engagement [2]. Based on the above theories and existing research, this paper intends to construct the following analysis model (Fig 1).

## 4 Research design

### 4.1 Data sources

Based on the compilation and analysis of relevant domestic and international literature, this paper has preliminarily developed the Survey Questionnaire on Undergraduates’ Willingness to Pursue Postgraduate Studies. To ensure the rationality and feasibility of the questionnaire options, the research team invited scholars in related fields and undergraduate students to discuss the questionnaire’s conceptual framework, indicators, and question types, thereby enhancing the survey’s validity. Following the initial drafting, revisions were made incorporating expert feedback, ultimately finalizing the questionnaire required for the investigation.

The target population for this study comprises full time undergraduate students enrolled in regular higher education institutions between January and February 2025 from representative universities in mainland China, particularly senior students at a critical juncture of graduation decisions, pursuing postgraduate studies or entering the workforce. The survey population comprises undergraduate students from both Double First Class universities and non-Double First Class institutions.

Data collection employed a combined strategy of two stage sampling and convenience sampling. First, universities were categorized into three sub-populations based on the Ministry of Education’s Double First Class Initiative List, Double First Class University Institutions, Double First Class Discipline Institutions, and non-double First Class institutions. A random number of universities were selected from each stratum to ensure the sample covered the primary types within China’s higher education system. Building upon this foundation, sampling quotas were established to achieve a roughly equal proportion of samples from Double First Class and non-double First Class institutions, thereby enhancing the structural representativeness of the sample. During the individual sampling phase, questionnaires were distributed through multiple channels including the email outreach and social networks. Emphasis was placed on covering diverse academic years and disciplines to obtain a varied sample.

The study protocol was reviewed and approved by the Research Ethics Review Board of Sichuan Normal University (Approval No. SNUE 2025LS0031). Prior to the commencement of the study, participants were made aware of the study in writing and made clear that the data were to be used for academic research only to ensure informed consent. Participation is entirely voluntary, and participants have the right to withdraw at any time.

The study collected a total of 1,347 questionnaires. After excluding invalid responses, 1,178 valid questionnaires were obtained, yielding a validity rate of 84.18%. The final sample comprised 221 students from top tier universities, 477 students from universities with top tier disciplines, and 480 students from non-double First Class universities, with the distribution of university types conforming to the quota design. Although non-probability sampling was employed, the sample size (N = 1178) exceeded the standard typically required for regression analysis. Post-hoc statistical power analysis also indicated that the current sample could detect small to medium effects (Power>0.99). The sample exhibited good diversity across key variables (Table 1), meeting the requirements for subsequent analysis (Table 2).

**Table 1:** Basic characteristics of the sample.

Personal Information	gender		Parental education				
	man	woman	Junior high school and below	High school or technical secondary school	post-secondary education (Specialist)	undergraduate	graduate student
undergraduate	867	311	400	320	158	260	40
Proportion (Percentage)	73.60%	26.40%	33.96%	27.16%	13.41%	22.07%	3.40%
Education Institute (University Condition) status	Types of university levels						
	Top Universities			Universities with First Class disciplines		non-double First Class universities	
undergraduate	221			477		480	
proportion	18.76%			40.49%		41.75%	
individual Information	Household registration		Monthly living expenses				
	town	countryside	0-700	700-1400	1400-2000	2000-4000	4000and above
undergraduate	663	515	23	415	452	245	43
proportion	56.28%	43.72%	1.95%	35.22%	38.37%	20.79%	3.67%

Note: although the sample sizes of different university types vary, the coefficient cluster regression and hypothesis tests employed in this study are based on the standard errors of the estimates, which inherently incorporate sample size information. Therefore, comparisons of coefficients across sub-models are statistically valid and fair.

**Table 2:** Summary of PLS SEM model evaluation metrics.

Indicator Type	Indicator	Value	Standard	Conclusion
Fit Indicator	SRMR	0.068	<0.08	Good
Structural Model Explanatory Power	R² (Learning Engagement)	0.274	Above average	Informative
	R² (Postgraduate Willingness)	0.328	average	Informative
Aggregate Reliability	AVE (Dedication)	0.612	>0.5	Satisfactory
Combined Reliability	CR (Dedication)	0.841	>0.7	Satisfactory
Reliability Indicator	Cronbach’s α (Total Learning Engagement)	0.812	>0.7	Good

Note: All measurement models meet the external load standard (Loading > 0.6) and exhibit no significant multicollinearity (VIF < 5).

### 4.2 Measurement and data analysis

#### 4.2.1 Family capital.

In the study of family capital, family capital is measured by the type of occupation of the parents and the annual household income, which is relatively broad and cannot explain the specific differences within the occupation [30]. In this study, family capital refers to the resources within the family that can support the student’s educational development. Following Bourdieu and Coleman, it includes financial capital economic resources, human capital parents’ education, and social capital family relationships available to the student. Thus, we measure family capital through three observed indicators including parents’ education level, average monthly living expenses provided or family financial investment, and relationship intimacy with parents. While each alone is not the entirety of family capital, together they represent the multidimensional family capital construct in research context.

#### 4.2.2 Learning engagement.

Learning engagement refers to the mental state of students who have full confidence and energy to face learning, and invest a lot of time and energy to actively learn after understanding the meaning of learning and getting the joy of learning, including learning vitality, learning dedication and learning concentration [31]. Each dimension of learning engagement corresponds to three questions in the questionnaire, and the answer results are divided into seven levels according to degree, among which the negative indicators have been converted into positive values, and the larger the value, the higher the degree of learning engagement (Table 3).

**Table 3:** Chinese revised version of the university work engagement scale-short form (UWES-S) developed by Schaufeli et al.

Item	1	2	3	4	5	6	7
1. Upon rising each morning, I am eager to study							
2. When studying, I feel full of energy							
3. Even when learning proves difficult, I remain undeterred and persevere							
4. I can study for extended periods without needing breaks							
5. When studying, I recover quickly from mental fatigue							
6. During study, I feel vigorous and highly motivated							
7. I find learning challenging							
8. Learning sparks my inspiration							
9. I am passionate about learning							
10. I take pride in my studies							
11. I find my learning purposeful and meaningful							
12. When studying, I forget everything around me							
13. Time flies when I am learning							
14. When studying, my mind is solely focused on the task at hand							
15. I find it difficult to put my studies aside							
16. I become completely absorbed in my studies							
17. When fully immersed in learning, I feel profoundly content							

#### 4.2.3 Willingness to go to graduate school.

Undergraduate students’ willingness to pursue postgraduate studies serve as the dependent variable in this research. Given the study’s focus on the core theme of domestic postgraduate entrance examinations in China, researchers have simplified the complex spectrum of post-graduation pathways into a binary variable. Namely, choosing to pursue postgraduate studies and direct employment. Here, choosing to pursue postgraduate studies refers to undergraduates planning to sit the national unified postgraduate entrance examination after graduation to continue their education at the postgraduate level. Direct employment constitutes a relatively broad category encompassing immediate employment, entrepreneurship, participation in civil service examinations, and other pathways not classified under pursuing postgraduate studies.

The measurement of learning engagement employed the Chinese revised version of the University of Waterloo Engagement Scale (UWES-S) developed by Schaufeli et al. This scale comprises vigor, dedication, and absorption, each measured by three items. All items were rated on a seven point Likert scale, anchored as 1 = “Strongly disagree”, 4 = “Neutral”, and 7 = “Strongly agree”. Higher scores indicate greater student learning engagement. This study made minor adjustments to the wording of the original scale to better align with the learning context of Chinese undergraduates. For reverse scored items within the scale, we performed standardized score conversion prior to data analysis to ensure all items were consistently oriented.

This research utilised Cronbach’s alpha to assess the assembled questionnaire before data processing. If the questionnaire’s alpha coefficient surpasses 0.8, it signifies outstanding reliability; an α coefficient ranging from 0.7 to 0.8 denotes relatively high reliability; an α coefficient between 0.6 and 0.7 implies acceptable reliability; an α coefficient below 0.6 suggests that the questionnaire or test results are suboptimal, requiring redesign and modification. The reliability analysis performed with SPSS 26.0 demonstrated that the majority of observed variables had reliability coefficients over 0.806, signifying that the questionnaire is very reliable. To reduce interference from extraneous variables and improve research reliability, this study designated gender, household registration status, and the postgraduate study experience of biological sisters as control variables for intentions regarding postgraduate examinations (Table 4).

**Table 4:** Classification and descriptive analysis of variables.

Variable type	variable	Mean	standard deviation	minimum	maximum	Description of the variable	Sources
Dependent Variable	Willingness to go to graduate school argument	0.736	0.441	0	1	0 = Direct employment; 1 = Take postgraduate entrance examination	[13]
	The average monthly cost of college students	1.890	0.879	1	5	1 = 0–700Yuan/Month; 2 = 700–1400 Yuan/Month; 3 = 1400–2000 Yuan/Month; 4 = 2000–4000 Yuan/Month; 5 = 4000Yuan and above/Month	[32]
	Parental education	12.514	3.062	9	22	According to the highest education of both parents, the number of years of education is converted, junior high school and below is converted into 9 years, and high school or secondary vocational secondary school, higher vocational college, bachelor’s degree, master’s degree and doctoral degree are converted into 12, 15, 16, 19 and 22 years in turn	[33]
	Intimacy with his father	2.518	0.589	1	3	1 = alienate; 2 = kind; 3 = intimacy	self-compiled
	Intimacy with mothers	2.738	0.468				self-compiled
	Learning engagement	3.653	1.637	1	7	The questionnaire options are divided into 7 levels according to the degree of agreement, and the negative indicator has been changed to a positive indicator. 1 = Lowest learning engagement;7 = Highest learning engagement	[31]
	Learning dedication	3.89	1.768				
	Learn focus	3.497	1.534				
Control variables	Gender	0.736	0.441	0	1	0 = woman; 1 = man	self-compiled
	Household registration	0.437	0.496	0	1	0 = Urban; 1 = Rural	self-compiled
	Graduate school experience of biological sisters	0.289	0.454	0	1	0 = No; 1 = Yes	self-compiled
	Whether it belongs to the experimental class	0.882	0.323	0	1	0 = No; 1 = Yes	self-compiled

#### 4.2.4 Data analysis.

Data were mainly analyzed by SPSS 26 for Windows, under a license held by university. The PROCESS macro 4.1 was used within SPSS to conduct the bootstrapped mediation analysis. Additionally, all graphs were produced with SPSS and Microsoft Excel.

To examine whether “learning engagement” mediates the relationship between “family capital” and “willingness to take postgraduate school entrance exam”, this study employs Bootstrap sampling and Monte Carlo simulation to test the mediating effect. Compared to traditional Sobel tests, these methods do not require the product term of the mediating effect coefficient to follow a normal distribution and exhibit higher statistical power. For Bootstrap method, this study drew 5,000 Bootstrap samples to generate estimates of the mediating effect and its 95% confidence interval. If the confidence interval did not include zero, the mediating effect was considered significant. For Monte Carlo method, this study also employed 5,000 simulations to generate more precise confidence intervals for the mediating effect, serving as a robustness check for the Bootstrap results. Table 7 presents the mediating effect analysis results for learning commitment across different university types, controlling for variables such as gender and household registration.

### 4.3 Analytical model

The dependent variable postgraduate entrance exam intention in this study is a dichotomous variable (0 = Direct employment; 1 = Take postgraduate entrance examination), thus a binary Logit regression model was employed for analysis. In the baseline specification, family capital (FC) and learning engagement (LE) are each composed of multiple secondary indicators. To clearly express the variable structure and comprehensively evaluate their overall effects, this study constructs the following two level model. First, a Logit regression model incorporating all secondary indicators was established:


$$\text{ln}\left(\frac{P_{i}}{\text{1}-P_{i}}\right)=\alpha +\sum_{j=\text{1}}^{m}\beta_{j}FC_{ij}+\sum_{k=\text{1}}^{n}\gamma_{k}LE_{ij}+\theta X_{i}+\varepsilon_{i}$$ (1)

Here, P_i_ denotes the probability that individual i chooses to pursue postgraduate studies; FC_ij_ represents the jth secondary indicator of family capital, while LE_ik_ denotes the kth secondary indicator of learning commitment; X_i_ accounts for other control variables; β_j_ and γ_k_ are the regression coefficients for secondary indicators, respectively; α is the intercept term, and ε is the error term.

This study employs the Coefficient Clustering Method to thoroughly assess the overall significance of family capital and learning engagement on intentions for postgraduate entrance examinations, while also tackling the difficulty of comparing effects across several indicators. This method aggregates the coefficients of secondary indicators across dimensions into a single primary composite indicator:


$${Effect}_{Cluster}=\frac{\sum_{j=\text{1}}^{k}w_{j}\beta_{j}}{\sum_{j=\text{1}}^{k}w_{j}}$$ (2)

Effect_cluster_ represents the cluster effect value for family capital or learning engagement intensity, where β_j_ denotes the regression coefficient for the jth variable in this variable cluster, w_j_ is its corresponding weight, and k is the number of variables included in this cluster. Subsequently, the cluster effect value can be substituted into the following comprehensive comparison model:


$$\text{ln}\left(\frac{P_{i}}{\text{1}-P_{i}}\right)=\alpha^{\prime }+{\lambda_{\text{1}}Effect}_{Fc}+{\lambda_{\text{2}}Effect}_{LE}+\theta^{\prime }X_{i}+\varepsilon_{i}^{\prime }$$ (3)

This model is used to directly compare the relative influence of two primary dimensions, family capital and learning engagement, on graduate school entrance exam intentions. λ_1_ and λ_2_ respectively reflect the direction and strength of their overall effects.

On the basis of the existing exploration of family capital and willingness to go to graduate school, and learning engagement and willingness to go to graduate school, this paper will also explore the relationship between family capital and learning engagement, and test the mediating effect of study engagement on the relationship between family capital and graduate school entrance examination intention.

## 5 Conclusion and discussion

### 5.1 The benchmark returns of undergraduate students’ willingness to go to graduate school

A step by step regression method was used to statistically analyze the variables of family capital, study engagement and willingness to enter graduate school (Table 5). In order to avoid the influence of the collinearity problem on the accuracy of the regression results, firstly, the regression equations of model 1, model 2, model 3 and model 4 were collinearity diagnosis. It is found that the VIF in each regression equation is less than 3, which can be concluded that there is no serious multicollinearity problem between the respective variables in this paper.

**Table 5:** Analysis of the influencing factors of undergraduate students’ willingness to go to graduate school.

	Equation (1)	Equation (2)	Equation (3)	Equation (4)
	All university samples	A sample of first class universities	Universities with first class disciplines	Non-“double first class” universities
Statements (argument)				
Household capital				
Monthly living expenses	0.198** (0.093)	0.446** (0.193)	0.055** (0.023)	0.133*** (0.046)
Parental education	0.021** (0.009)	−0.042** (0.018)	−0.037** (0.015)	0.034*** (0.011)
Relationship with father	−0.129** (0.053)	0.001** (0.000)	−0.179** (0.075)	−0.151** (0.062)
Relationship with mother	−0.357*** (0.089)	−0.420*** (0.135)	−0.403*** (0.141)	−0.308*** (0.106)
Learning engagement				
Learning vitality	0.014* (0.007)	0.042* (0.021)	−0.009* (0.005)	0.047* (0.025)
Learning dedication	0.114** (0.053)	0.029*** (0.007)	0.059** (0.022)	0.186** (0.089)
Learn focus	0.074** (0.032)	0.078*** (0.027)	0.063** (0.027)	0.072** (0.032)
Control variables				
gender	0.067** (0.029)	−0.242** (0.107)	0.215** (0.095)	0.320** (0.132)
Household registration	−0.507*** (0.178)	−1.204** (0.568)	−1.213*** (0.336)	−0.039** (0.016)
Biological sister college experience	−0.154* (0.086)	0.420* (0.221)	−0.346** (0.150)	−0.137* (0.075)
Whether it belongs to the experimental class	1.093*** (0.309)	1.292*** (0.417)	0.989** (0.437)	0.801*** (0.243)
Expert (specialized)	control	Control	control	control
Sample size	1178	221	477	480
Log likelihood	−626.657	−78.916	−207.492	−298.314
Pseudo R2	0.078	0.137	0.113	0.070

Note: Values in parentheses are standard deviations; ***、**、* Respectively indicated in 1%、5%、10% horizontally significant, the same as in the table below.

Secondly, the control variables, gender, household registration, biological sisters’ college experience, whether they belong to experimental classes, etc., and the independent variables family capital, monthly living expenses, parents’ education level, relationship with parents, etc., and learning engagement, learning vitality, learning dedication, and learning monograph, were substituted into regression model 1, model 2, model 3, and model 4, respectively, to explore the differences in the influence of independent variables on students’ willingness to enter graduate school in different types of colleges.

Through benchmark regression analysis, research hypothesis 1 was validated. (1) The graduate studies experiences of biological sisters significantly influenced undergraduates’ willingness to pursue graduate education. At First Class universities students are 1.52 times more likely to choose to go to graduate school because their biological sisters have gone to graduate school ($e^{0.42}$ ). Bandura’s social learning theory believes that in the process of forming their own concepts and behaviors, individuals will take the surrounding groups as role models, learn from the behavior of role models, and finally form their own behavior patterns. Undergraduate students in First Class universities are influenced by their surroundings or relatives when they choose to go to graduate school, and when their biological sisters actively choose to study in graduate school, college students will move closer to their biological sisters and choose to study in graduate school [34]. However, in the sample of First Class universities and non-double First Class universities, the graduate study experience of biological sisters will reduce the probability of undergraduate students entering graduate school, which may be due to the resource dilution theory, which believes that the number of college students in the family is negatively correlated with the number of undergraduate students’ education years under the constraints of given family resources [35]. Downey and Condron (2004) and Blake (1981) explains that specifically, under the condition of family resources, siblings continue to receive graduate education, which occupies more family resources, making it impossible for families to provide high quality family resources for undergraduates [36]. At this time, the willingness of undergraduates to enter graduate school changed as their siblings continued to study in graduate school.

(2) Monthly living expenses exhibit a significant positive correlation with undergraduates’ willingness to pursue postgraduate studies. Logistic regression results indicate that when living expense levels rise by one interval, the odds ratio for the occurrence of postgraduate study intentions is approximately 1.22 times (*e*^0.198^–1) the original value. Maslow’s Hierarchy of Needs Theory believes that survival needs are the basis for individuals to pursue other needs, and when individual survival needs are satisfied, they will pursue higher level needs. The monthly living expenses of undergraduates are the basis of undergraduates’ school life, and when the monthly expenses can meet the material needs of undergraduates, undergraduates have the time and energy to pursue higher level needs. From the perspective of different types of universities, the proportion of First Class universities increasing the likelihood of increasing graduate studies is higher than that of First Class universities and non-double First Class universities for each increase in monthly living expenses, which may be due to the fact that students in First Class universities have a stronger willingness to study in graduate school, and the marginal utility of material security to bring higher level demand is higher than that of students from First Class universities and non-double First Class universities.
(3) The influence of parental education duration on undergraduate students’ inclination to continue postgraduate degrees is variable and demonstrates variation among various university kinds. On average, each additional year of parental education increases the likelihood of undergraduates choosing to pursue postgraduate studies by 2.12% ($e^{0.021}-1$), but this growth effect is primarily concentrated in non-double First Class universities. Conversely, at double First Class universities, higher parental education levels actually reduce students’ willingness to pursue postgraduate studies. This may stem from the fact that parents of students at these institutions generally possess higher educational backgrounds, hold greater expectations for their children’s education, and are more inclined to encourage overseas study. Furthermore, double First Class institutions possess greater resource advantages in terms of facilities, faculty strength, graduate school admission quotas, study abroad applications, and graduate summer camps. They also disseminate more extensive information about further education. These institutional resources partially substitute for the function of family cultural capital, thereby weakening the direct influence of parental education levels on graduate school aspirations. Notably, within top tier universities, family cultural capital exhibits a “ceiling effect”, where high parental educational attainment becomes the norm, diminishing its marginal utility. In such institutions, the roles of institutional platforms and individual agency become more prominent. Conversely, at non-double First Class universities with relatively limited resources, students demonstrate stronger dependence on family capital.

### 5.2 Cluster regression of the degree of influence of independent variables

The cluster regression results of model 1, model 2, model 3 and model 4 were obtained by using the coefficient cluster method, and the cluster regression results of model 1, model 2, model 3 and model 4 were obtained (Table 6).

**Table 6:** Comparison of the effect values of family capital and study engagement on undergraduate students’ willingness to enter graduate school.

		Equation (1)	Equation (2)	Equation (3)	Equation (4)
		All university samples	A sample of First Class universities	Universities with First Class disciplines	Non-“double First Class” universities
Post-mortem estimate 1	Household capital	0.287** (0.119)	0.373*** (0.120)	0.273** (0.121)	0.262* (0.139)
	Learning engagement	0.461*** (0.121)	0.732*** (0.153)	0.512*** (0.128)	0.346** (0.150)
Post-mortem estimate 2	Household capital				
	Monthly living expenses	0.253** (0.110)	0.350*** (0.117)	0.105** (0.045)	0.164** (0.071)
	The level of education of the parents	0.137*** (0.050)	0.132*** (0.051)	0.082*** (0.028)	0.153*** (0.049)
	Closeness with parents	0.218** (0.089)	0.184** (0.080)	0.241* (0.134)	0.171* (0.097)
	Learning engagement				
	Learning vitality	0.059** (0.026)	0.107*** (0.030)	0.047** (0.020)	0.098*** (0.031)
	Learning dedication	0.400*** (0.101)	0.618*** (0.215)	0.258*** (0.068)	0.310** (0.143)
	Learn focus	0.180** (0.083)	0.258** (0.121)	0.199*** (0.073)	0.158** (0.072)

A horizontal comparison of the influencing factors of each sample shows that: (1) The higher the level of the university, the stronger the effect of family capital on the willingness of undergraduates to enter graduate school. On the one hand, the influence of family capital on students is no longer limited to the college entrance examination, and it also influences undergraduates’ inclination to pursue graduate studies. On the other hand, it shows that the effect of family capital is stronger with the higher level of universities, which also confirms the role of Bourdieu’s cultural reproduction theory in the accumulation of human capital during the university period.

**Fig 2:**
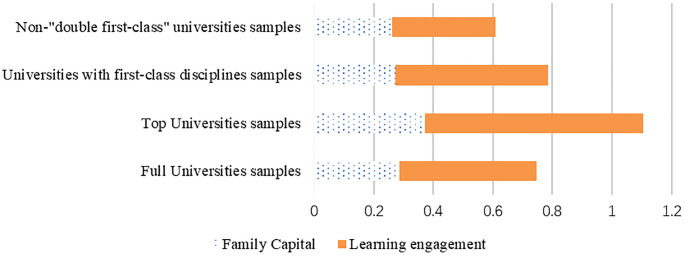
Comparison of the relative importance of household capital and learning engagement.

(2) Research hypothesis 3 was validated. The influence of learning engagement, the primary effect, is substantially more pronounced than that of family capital. Both family capital and learning engagement influence undergraduate students’ willingness to pursue postgraduate studies, but learning commitment exerts a stronger influence than family capital. As shown in Post-hoc Estimate 1, in Models 1–4, learning commitment is 1.606 times, 1.962 times, 1.875 times, and 1.321 times that of family capital, respectively. When the combined influence of academic commitment and family capital is constrained to 1, the results show that academic commitment accounts for approximately 60% of the variance in each model, while family capital’s influence remains between 33% and 44% (Fig 2). This core finding strongly supports Boudon’s theoretical distinction, indicating that at the higher education stage, the primary effect of academic performance achieved through individual effort begins to surpass the direct influence of secondary effects from family background in college admission decisions. This does not imply that family capital becomes irrelevant, but rather that its mode of operation becomes more indirect by influencing learning commitment. This provides empirical evidence for the performance selection theory in contemporary China, demonstrating that higher education continues to play a crucial role in promoting social mobility.

(3) Research hypothesis 2 was validated. The higher the level of colleges and universities, the higher the students’ learning engagement. As the university level increases, so does the vitality, dedication, and concentration of undergraduates, with learning engagement exerting the most pronounced influence. According to the analysis of the results of Post-hoc estimation 2, the degree of influence of learning dedication is 6.78 times and 2.22 times that of learning vitality and learning concentration, respectively, and it is also 1.43–1.83 times that of family capital of different types of universities. During the period of university study, the more undergraduate students who study and dedicate themselves, the stronger the willingness to choose to go to graduate school. In addition, there are also differences in the degree of influence of learning dedication on the willingness of students in different types of universities to enter graduate school, and the level of learning dedication significantly influences the willingness to pursue graduate studies among undergraduates from First Class universities and those from non-double First Class universities.

### 5.3 Analysis of the mediating effect of learning engagement

In order to explore the mediating role of learning engagement on the relationship between family capital and willingness to go to graduate school, this study intends to use the mediating effect method of Bootstrap and Monte Carlo to analyze. On the one hand, these two methods can accurately analyze the non-orthometric distribution data, and on the other hand, they can compare and test the effects of multiple mediation paths [37]. Table 7 presents the mediating role of learning engagement in different types of colleges and universities on the basis of controlling for demographic variables such as students’ gender and household registration. And research hypothesis 4 was validated.

**Table 7:** The mediating role of learning engagement in different types of universities.

Sample	Mediation effect	coefficient	Standard error	Distinctiveness	95% Bootstrap Confidence Interval	Monte Carlo Confidence Interval
Total Sample	family capital – learning engagement – willingness to go to Graduate School	0.093	0.036	Significant	[0.024, 0.162]	[0.021, 0.157]
First Class universities	family capital-study engagement-willingness to go to graduate school	0.132	0.029	Significant	[0.076, 0.192]	[0.071, 0.189]
First Class discipline	family capital-learning engagement-willingness to go to graduate school	0.114	0.031	Significant	[0.053, 0.181]	[0.050, 0.179]
Non-double First Class universities	family capital-learning engagement-willingness to go to graduate school	0.076	0.018	Significant	[0.037, 0.113]	[0.035, 0.110]

The analysis of the mediating role of learning engagement shows that the mediating role of learning engagement not only exists in the whole sample, but also plays a role in the samples of different levels of colleges and universities. Specifically, the mediating role of learning engagement is also different among the different levels of universities, and the influence of family capital on undergraduates’ willingness to go to graduate school is greater than that of non-double First Class universities. This reflects that family capital indirectly affects undergraduates’ willingness to go to graduate school by influencing their learning engagement, and the degree of indirect influence of family capital on undergraduates’ willingness to go to graduate school is also different depending on the type of university.

Bandura’s social learning theory posits that individuals form their own beliefs and behaviors by observing and emulating role models within their social groups. Undergraduate students at top tier universities are influenced by their surroundings or relatives when deciding whether to pursue postgraduate studies. When biological sisters actively choose to pursue graduate education, undergraduates tend to align with their sisters’ choices and opt for graduate studies as well. However, in samples from top tier discipline universities and non-double First Class institutions, having biological sisters pursue graduate studies reduces undergraduates’ likelihood of doing so themselves. This may stem from the resource dilution theory, which posits that within fixed family resource constraints, the number of children in a household correlates inversely with the total years of education received by those children. This finding strongly corroborates Goldthorpe’s relative risk avoidance theory. For students at top tier universities, whose families occupy higher socioeconomic strata, the motivation to avoid downward mobility is stronger. When biological sisters pursue graduate studies, they not only provide a role model but also send a powerful signal: maintaining family status requires higher academic credentials. Consequently, their motivation to pursue graduate studies significantly increases. Conversely, for students at other universities, family resources may be more limited. In this context, the resource dilution theory becomes a practical constraint on risk aversion: the allocation of family resources among multiple students’ education forces them to engage in more pragmatic cost benefit calculations during decision making, thereby weakening their motivation to pursue postgraduate studies. This reveals the conditional nature of family capital’s influence: when resources are abundant, it acts as a driving force enhancing motivation; when resources are constrained, it functions as a limiting constraint on choice.

## 6 Limitation and implication

The present study illuminates how family capital and undergraduates’ learning engagement jointly shape students’ intentions to pursue postgraduate education. Findings indicate that family capital is a significant positive predictor of the intention to take the postgraduate entrance examination. Students from families with greater economic and cultural resources were more inclined to plan for postgraduate studies, underscoring the enduring influence of family background on educational aspirations. Learning engagement also emerged as a strong, direct facilitator of postgraduate entrance exam intentions, students who invested more time and effort in their academic work and campus learning opportunities reported higher motivation to continue their education. Importantly, learning engagement was identified as a key mediating mechanism linking family capital to postgraduate intentions. In research analyses, family capital fostered higher learning engagement, which in turn translated into stronger intentions to pursue a graduate degree. This mediated pathway suggests that family advantages are partly channeled through students’ learning engagement, consistent with educational reproduction theories. Even after accounting for this mediation, family capital retained a partial direct effect on intentions, indicating that both family resources and personal engagement independently contribute to the decision to undertake further study.

### 6.1 Limitation

This study examines the relationship between family capital, academic engagement, and undergraduate students’ willingness to pursue postgraduate studies by constructing an empirical model, yielding several meaningful findings. Despite its contributions, this study has several limitations that warrant acknowledgment.

First, regarding methodology, this study used cross-sectional data to reveal correlations and mediating mechanisms among variables. However, due to data limitations, establishing strict causal inferences remains challenging. While theoretical logic supports the pathway “family capital, academic engagement, postgraduate study willingness” reverse causality or confounding from omitted variables cannot be entirely ruled out. Future research may consider panel tracking or experimental methods to more clearly elucidate causal links and dynamic mechanisms among variables.

Second, all measures, including family capital indices, learning engagement, and intention to take the exam, were obtained via self-report questionnaires, raising the possibility of common method bias and subjective measurement error. Future studies might incorporate more objective indicators such as academic records or parental education data or multi-informant reports to strengthen validity.

Third, the sample was drawn from a specific population, which may limit generalizability. The participants may not fully represent all Chinese college students, and the effect of family capital might differ in other types of universities or regions.

Fourth, the cultural context of this study is unique, the postgraduate entrance examination is a highly competitive pathway in China, and family influence on education can be culturally specific. Caution should be taken in extending these conclusions to different educational systems or cultural settings without additional comparative research. Lastly, our focus was on intention as the outcome; while intention is a strong precursor to behavior, not all students who intend to take the exam will actually do so or succeed, and future research could track actual enrollment outcomes.

Furthermore, this study is subject to several additional limitations. For instance, in order to maintain research focus and model simplicity, graduation intentions were operationalized as a binary variable comprising “pursuing postgraduate studies” and “entering employment”. This approach failed to encompass other significant pathways such as studying abroad, starting one’s own business, or taking civil service examinations, thereby partially diminishing the information yielded by the diversity of graduation choices. Future research could expand the sample size and employ more flexible analytical methods, such as multinomial logit models, to conduct a more detailed classification and exploration of undergraduate graduates’ post-graduation destinations. It should also be noted that while the sampling method employed in this study covers multiple types of higher education institutions, it constitutes non-probability sampling. The sample exhibits a relatively high proportion of male students (73.6%) and those with urban household registration (56.28%), which may deviate from the actual composition of undergraduate students nationwide. This limitation may affect the generalizability of the study’s conclusions. It is recommended that subsequent research adopt probability sampling methods to enhance the representativeness of the sample.

### 6.2 Implication

These findings offer several practical and policy implications grounded in our theoretical framework. For validation and extension of the Boudon model. Empirical findings support the distinction between the “primary effect” and the “secondary effect”. However, further analysis reveals that in Chinese university students’ decisions to pursue postgraduate studies, a significant portion of the “secondary effect”, family capital, is mediated through its influence on the “primary effect”, academic engagement. This indicates that the two effects are not entirely independent but exhibit complex interactions, deepening our understanding of their underlying mechanisms.

For refining the context of risk aversion theory, the study reveals that the strength of family capital’s influence varies across university tiers. In top tier institutions, both the direct and indirect effects of family capital are more pronounced. This indicates that Goldthorpe’s relative risk aversion behavior is not static but embedded within specific institutional contexts, such as university hierarchy. Higher tier institutions offer clearer pathways to elite status, prompting students, and their families to more actively mobilize capital for risk avoidance, thereby reinforcing the reproduction of class advantages. This links macro level institutions with micro level individual behavior, broadening the explanatory scope of the theory.

For clarification of variable mechanisms, this study not only validates the effects of family capital and academic commitment but also quantifies their relative importance through coefficient clustering and reveals their causal pathways via mediation models. Notably, it identifies “close parent child relationships” as the most influential component of family capital and “academic dedication” as the critical factor within academic commitment, providing more refined variable measurements and research entry points for subsequent studies.

All in all, this study employs cross-sectional data to elucidate the relationships among variables and the mediating processes at the methodological level. The data’s limitations impede the establishment of definitive causal inferences. Theoretical logic indicates the sequence of “family capital → learning commitment → aspirations for postgraduate entrance examinations”. Nonetheless, reverse causality and confounding due to omitted variables cannot be completely excluded. Future research could utilize panel monitoring or experimental methods to more precisely identify causal relationships and dynamic mechanisms among variables.

This study enhances the understanding of the intersection between family resources and student engagement in shaping educational trajectories. This study illustrates that learning engagement serves a crucial mediating function in conveying the advantages of family capital to postgraduate aspirations, thereby elucidating the mechanism by which social background impacts academic trajectories. Our research identifies this mechanism, enriching theoretical discourse on status attainment and capital in education. It also highlights actionable avenues for fostering student engagement and addressing resource disparities, thereby supporting undergraduates in achieving their postgraduate ambitions. The insights presented establish a basis for additional research and practical initiatives aimed at ensuring that the choice to pursue graduate education is influenced by students’ potential and commitment, rather than limited by their family situations.
